# Comparison of image quality and in vivo appearance of the normal equine nasal cavities and paranasal sinuses in computed tomography and high field (3.0 T) magnetic resonance imaging

**DOI:** 10.1186/s12917-016-0643-6

**Published:** 2016-01-19

**Authors:** Joachim Kaminsky, Astrid Bienert-Zeit, Maren Hellige, Karl Rohn, Bernhard Ohnesorge

**Affiliations:** Clinic for Horses, University of Veterinary Medicine Hannover, Foundation, Bünteweg 9, Hannover, D-30559 Germany; Institute for Biometry, Epidemiology and Information Processing, University of Veterinary Medicine Hannover, Foundation, Bünteweg 2, Hannover, D-30559 Germany

**Keywords:** 3 Tesla, High field MRI, CT, Equine paranasal sinuses

## Abstract

**Background:**

Computed tomography (CT) is a well-established imaging technique in the diagnostics of equine sinunasal disease. High-field magnetic resonance imaging (MRI) is becoming more readily available in equine veterinary medicine. MRI is appreciated for its superior ability to depict soft tissues with high contrast. To compare the established technique of CT in the depiction of the equine nasal cavities, paranasal sinuses and adjoining anatomical structures to 3 Tesla MRI the nasal cavities and paranasal sinuses of 13 horses were examined using CT and 3 Tesla MRI.

**Results:**

Comparison of CT and MRI images of the paranasal sinuses, nasal cavities and adjoining anatomical structures of 13 healthy horses showed that the inter-rater agreement for the CT examinations was higher than the inter-rater agreement for the MRI examinations. CT images proved to be significantly higher rated for the depiction of cortical bone, while MR images were higher rated for the appearance of soft tissues. For the distinction between different tissues or anatomical structures the MR images were significantly higher rated and especially T2-weighted sequences allowed for a good distinction between delicate structures. None of the MRI sequences produced an exact depiction of the lumen of the nasomaxillary aperture while the CT with a bone window allowed for a satisfying visualization.

**Conclusion:**

The CT is an imaging modality that produces high quality images within a short time when examining equine nasal cavities and paranasal sinuses. The strength of CT lies in the high quality depiction of large and delicate structures with high radiodensity. High field MRI with a field strength of 3 Tesla produces images of high quality that allow for the distinction of delicate soft tissue structures but requires long examination times. The high field strength of 3 Tesla magnetic imaging introduces new possibilities in tomographic soft tissue imaging of the equine head but cannot match up with the CT in terms of visualization of bone and total examination duration. Therefore, clinicians should consider the exact imaging needs in clinical cases to decide whether a single examination or a combination of both imaging techniques may promise the greatest benefit for the patient.

## Background

Clinical diagnostics of the equine paranasal sinuses during pathology often require the use of an imaging technique to determine the cause and extent of the disease [1]. X-ray has proven to be a helpful tool in the diagnostics of equine paranasal disorders [1]. Nevertheless, superimposition of structures on x-ray images makes interpretation difficult and often inaccurate [2–6]. Tomographic imaging techniques abolish the disadvantage of superimposition and particularly CT has become a well-established imaging technique in the diagnostics of equine sinunasal disease due to its good bone to soft tissue contrast [7–13].

MRI is appreciated for its superior ability to depict soft tissues with high contrast [7]. High-field MRI of 1.5 Tesla is becoming more readily available in equine veterinary medicine [14–17] and even 3 Tesla MRI is starting to be used in equine veterinary diagnostics [18–20]. The higher field strength of 3 Tesla has only recently been introduced in equine veterinary medicine and opens up new possibilities but also bears disadvantages and challenges when compared to MRI of lower field strengths [18–20].

The aim of this study was to compare the established technique of CT in the depiction of the equine nasal cavities, paranasal sinuses and adjoining anatomical structures to 3 Tesla MRI in healthy horses. This work provides suggestions for which anatomical structures the MRI or rather the CT should be the technique of choice, and elucidates whether or not the high field strength of 3 Tesla MRI has benefits when compared to CT.

## Methods

### Study population

Thirteen healthy horses of different breeds (7 Warmbloods, 2 Arabian thoroughbreds, 3 Standardbreds, 1 Andalusian) were used to acquire the CT and 3 Tesla MR images. The group was composed of 8 mares, 4 geldings and 1 stallion. The age ranged from 4 to 20 years (mean ± SD, 11.6 ± 6.5). All horses showed no signs of disease related to the nasal cavities or the paranasal sinuses. Image acquisition was performed with horses under general anaesthesia. This study was approved by the Lower Saxony State Office for Consumer Protection and Food Safety’s ethic committee. File reference: 33.9-42502-04-11/0592.

### Computed tomography

For the CT examination the horses were positioned in right lateral recumbency on a stationary examination table. CT was performed using a Brilliance™ CT – Big Bore Oncology Scanner (Philips Medical Systems, Best, The Netherlands).

The entire head of each horse was scanned with an axial scan-mode with 140 kV, 500 mAs, a slice thickness of 1.5 mm and an image matrix of 1024. Using multiplanar reconstruction, two transverse image sequences, one with a bone window (CTbw; WL: 300, WW: 2800), and one with a soft tissue window (CTstw; WL: 50, WW: 500) were generated. Both sequences were oriented perpendicular to the hard palate.

### Magnetic resonance imaging

**Fig. 1:**
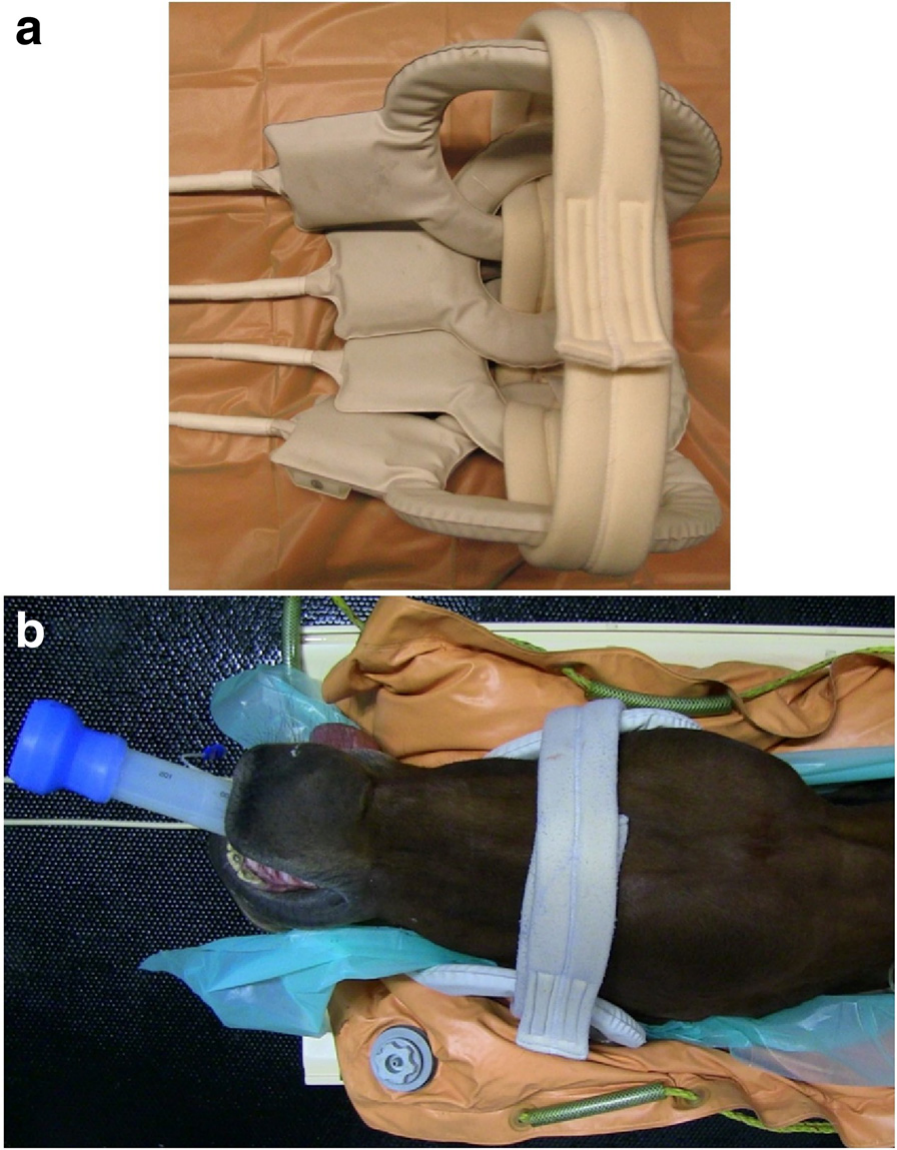
Head positioning for MRI examination. **a** Surface coils arranged for positioning (Philips SENSE™ Flex M® and Philips SENSE™ Flex L®; Philips Medical Systems, Best, The Netherlands); **b** Head positioned on the examination table with surface coils placed around the ROI

Subsequently to the CT examination the horses were positioned in dorsal recumbency on a non-stationary table and cushioned by an inflatable mattress for the duration of the examination. Surface coils (Philips SENSE™ Flex M® and Philips SENSE™ Flex L®; Philips Medical Systems, Best, The Netherlands) were positioned around the sinunasal region and the head was fixed in position with a vacuum cushion (Fig. 1).

Magnetic resonance imaging was performed using a Philips Achieva™ 3.0 T X-Series® (Philips Medical Systems, Best, The Netherlands). Sequences were obtained in transverse planes orientated perpendicular to the hard palate and included T2 weighted turbo spin echo (T2w) (TR: 4500 ms, TE: 80 ms, NSA: 2, ST: 4.0 mm; ISS: 1.0 mm, matrix: 1024 x 1024) and proton density weighted turbo spin echo (PDw) (TR: 8300 ms, TE: 30 ms, NSA: 2, ST: 4.0 mm; ISS: 0.4 mm, matrix: 960 x 960) sequences. T1 weighted gradient echo (T1w) images (TR: 8 ms, TE: 4 ms, NSA: 2, ST: 0.9 mm; ISS: 0 mm, matrix: 720 x 720) were obtained as a 3-dimensional (3D) -dataset with isotropic voxels so that transverse planes could be reproduced using multiplanar reconstruction. MRI settings were chosen to gain maximum image quality within a reasonable time frame. In all horses and sequences it was attempted to picture the horse’s head from a transverse line through the eyes to a plane right rostral to the first molar tooth.

The durations of the CT and MRI examinations, including the positioning of the horse on the examination table, as well as the time required for each MRI sequence were recorded. Additionally, the entire time of general anaesthesia was recorded.

### Image interpretation

**Fig. 2:**
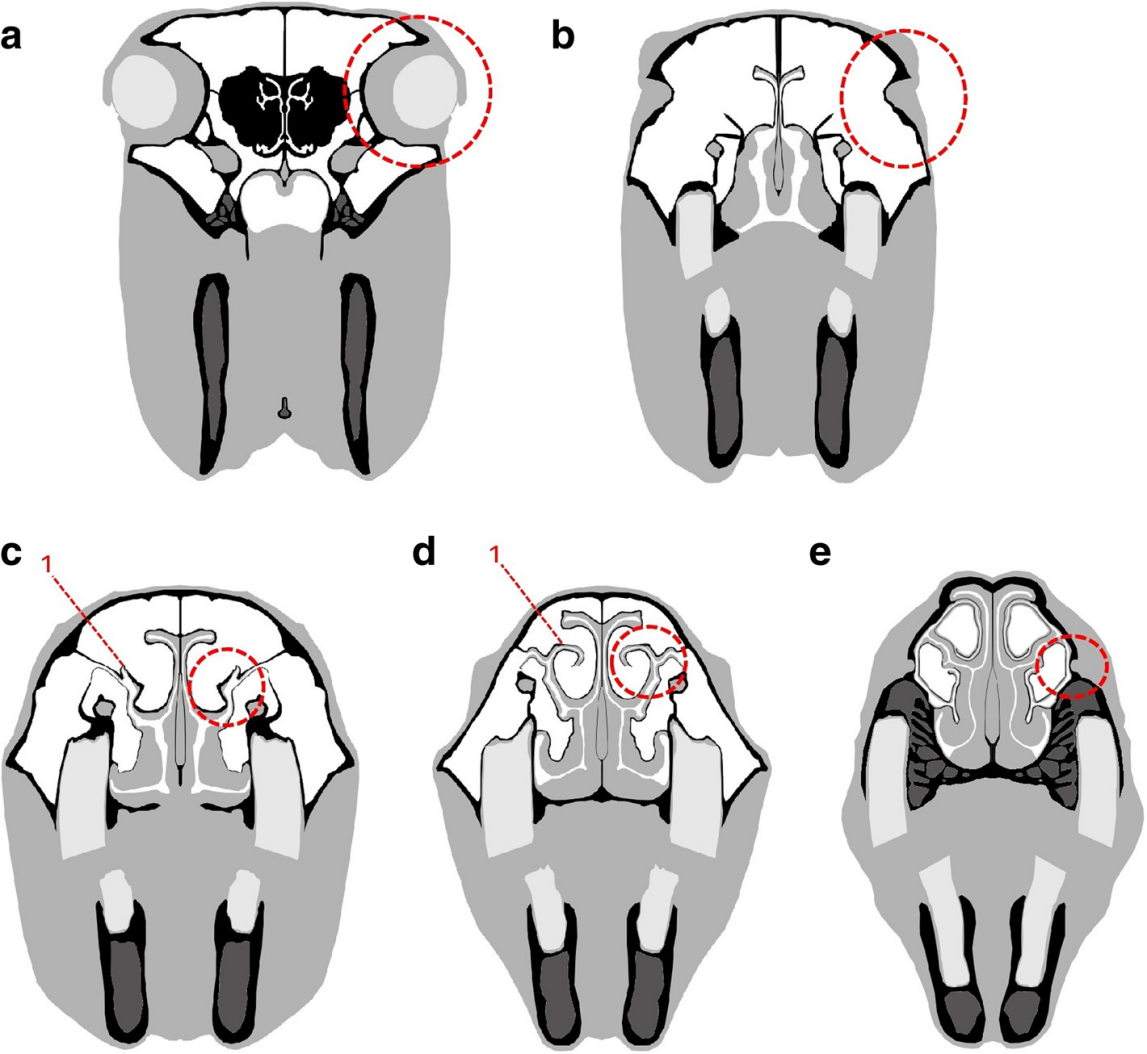
Schematic images of chosen planes. **a** to **e** Dashed circle indicates the point of orientation. **a** Plane 1 positioned on a plane through the centre of the ocular bulbs; **b** Plane 2 positioned immediately rostral to the eyes; **c** Plane 3 positioned on the level of the nasomaxillary aperture, a ‘hook’ (red 1) [38] protruding dorsally from the spiral lamella of the dorsal concha in all horses was used as an anatomical landmark; **d** Plane 4 positioned on the level of the nasomaxillary aperture, where the ‘hook’ (red 1) [38] starts to extend mediodorsally; **e** Plane 5 positioned on the level of the infraorbital foramen

After acquisition of CT and MR images slices from different planes of the head were chosen. Five slices from each imaging technique and each horse were selected. To ensure comparability of the selected slices, predefined anatomical landmarks were used. The anatomical landmarks are shown in Fig. 2 (a-e).

The chosen images were evaluated by three independent veterinarians with experience in CT and MRI image evaluation. The evaluation was performed under the same conditions for all three observers. For image interpretation three DICOM certified TFT displays (EIZO FlexScan MX190S; EIZO Europe GmbH, Mönchengladbach, Germany and the imaging software eFilm Workstation® 1.5.3 (eFilm Medical, Toronto, Canada) were used. Observers were allowed to alter the window level and window width in all MRI sequences, but were not allowed to change level presets in the CT images so that image characteristics remained unaltered. Images were evaluated for general image quality characteristics (Table 1).

**Table 1:** Scoring system for the image quality parameters

Score	Image noise	Image sharpness	Image contrast
0 - poor quality	very high image noise level	low image sharpness	low image contrast
1 - moderate quality	high image noise level	moderate image sharpness	moderate image contrast
2 - satisfying quality	moderate image noise level	satisfying image sharpness	satisfying image contrast
3 - good quality	no image noise	high image sharpness	high image contrast

For each plane a set of anatomical structures, tissues or tissue borders was defined. In each of the chosen anatomical structures, tissues or tissue borders scores were given for contour distinction, tissue distinction or detail resolution (Table 2). All five imaging techniques were compared and evaluated in a graded score system between 0 and 3 with 0 representing poor and 3 representing excellent performance. Observers were provided with a scoring guideline stating the anatomical structure and the property that was to be rated for this particular anatomical structure.

**Table 2:** Scoring system for the anatomical parameters

Score	Contour distinction	Tissue distinction	Detail resolution
0	not distinguishable	not distinguishable from each other	no details
1	distinguishable but often blurred	difficult to distinguish from each other	low details
2	well distinguishable and seldom blurred	well distinguishable from each other	high details
3	very well distinguishable and sharply defined	very well distinguishable from each other	excellent details

For simplification the different evaluated anatomical parameters were pooled for similar characteristics and sorted into four groups as shown in Table 3.

**Table 3:** Groups of evaluated anatomical parameters

Group	Characteristics	Anatomical parameters
1	soft tissue	venous plexus of the nasal mucosa
		palatine artery
		soft tissue inside the infraorbital canal
2	contour distinction of cortical bone	frontal and nasal bone plate
		vomer
		infraorbital canal
3	anatomical detail	cancellous bone
		ethmoid bone
		lumen of the nasomaxillary aperture
4	tissue distinction	distinction between air and mucosal lining of the rostral maxillary and conchofrontal sinus
		distinction between mucosal lining and cortical bone of the rostral maxillary and conchofrontal sinus
		distinction between venous plexus lining the nasal septum and cortical bone or cartilage
		distinction between bone and cartilage of the vomer and nasal septum

### Statistical analysis

Data collection was performed in spreadsheets using the software Office Excel 2003® (Microsoft Corporation, Redmond, WA, USA). For statistical analysis the software SAS® 9.3 (SAS Institute, Cary, NC, USA) was used.

Descriptive statistics were performed. Differences in inter-rater agreement were analysed using the McNemar-Bowker test of internal symmetry and calculating the Cohen’s kappa coefficient.

To determine the variations between the five different imaging techniques a permutation test with 1000 simulations was performed for each evaluated parameter and between each technique. A *P* value <0.05 was considered statistically significant.

## Results

A total of 13 horses were examined. In all horses the combined examination of CT and MRI was performed without complications. In the CT examination the entire head of each horse was scanned. The field of view in the MRI examination from caudal to rostral for all horses ranged from 217 to 300 mm (mean ± SD; 245.1 mm ± 27.0 mm). CT examinations took 10 to 18 min (mean 15 min) including positioning of the horse. MRI examinations took 61 to 71 min (mean 67 min) including positioning of the horse (Table 4).

**Table 4:** Range of duration for the examination parts

Part of examination	Range of duration (min)	Mean duration (min)
total time of general anaesthesia	76–89	84
CT examination (incl. positioning)	10–18	15
T2w	20–26	24
T1w	12–14	13
PDw	19–23	21
MRI examination (incl. positioning)	61–71	67

Overall 325 images were evaluated and 702 parameters were graded by each evaluator.

### Soft tissues

**Fig. 3:**
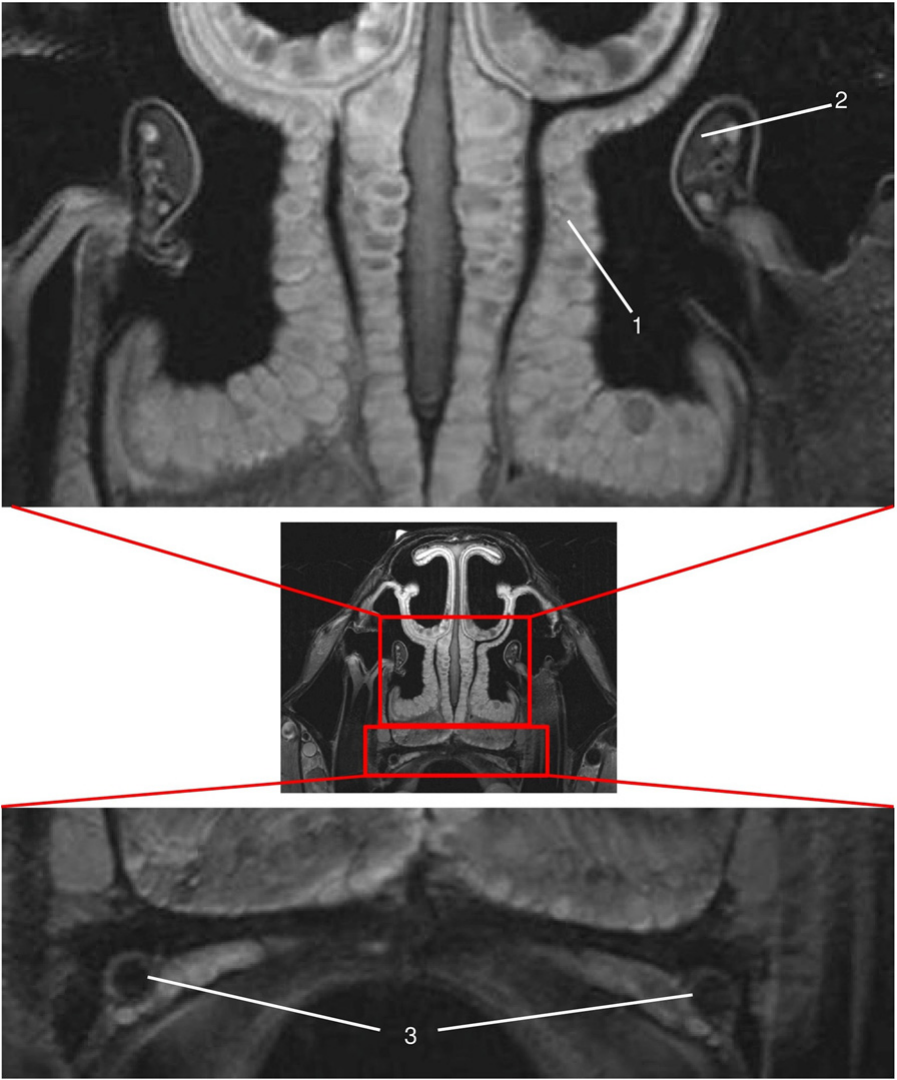
T2w transversal image in plane 3. (1) venous plexus of the nasal mucosa, (2) infraorbital nerve, (3) palatine arteries

T2w was significantly superior to all other imaging techniques in displaying the venous plexus of the nasal mucosa (Fig. 3), whereas the palatine artery and the soft tissue inside the infraorbital canal were displayed with no significant difference in quality by T2w (Fig. 3) and PDw. T1w was the lowest rated MRI sequence in all three compared locations. When comparing CT to MR imaging, CTbw and CTstw were significantly lower rated than all MRI sequences, as no significant difference between the bone and soft tissue window was present (Table 5).

**Table 5:** Median of scoring results of all 3 evaluators for soft tissue appearance

Imaging technique	CTbw		CTstw		T2w		T1w		PDw		score
anatomical parameter	m	%	m	%	m	%	m	%	m	%	
venous plexus of the nasal mucosa	0	100	0	100	3	0	0	60.3	2	1.9	0
		0		0		7.7		39.1		39.7	1
		0		0		30.1		0.6		48.7	2
		0		0		62.2		0		9.6	3
palatine artery	0	100	0	100	2	0	1	3.8	2	0	0
		0		0		7.7		82.1		7.7	1
		0		0		44.9		14.1		56.4	2
		0		0		47.4		0		35.9	3
soft tissue inside the infraorbital canal	0	100	0	91.5	2	0	1	20.5	2	2.6	0
		0		8.5		11.1		65.8		18.8	1
		0		0		53.0		13.7		49.6	2
		0		0		35.9		0		29.1	3

m = median; % = distribution of awarded scores (0, 1, 2, 3) in percent

### Contour distinction of cortical bone

**Fig. 4:**
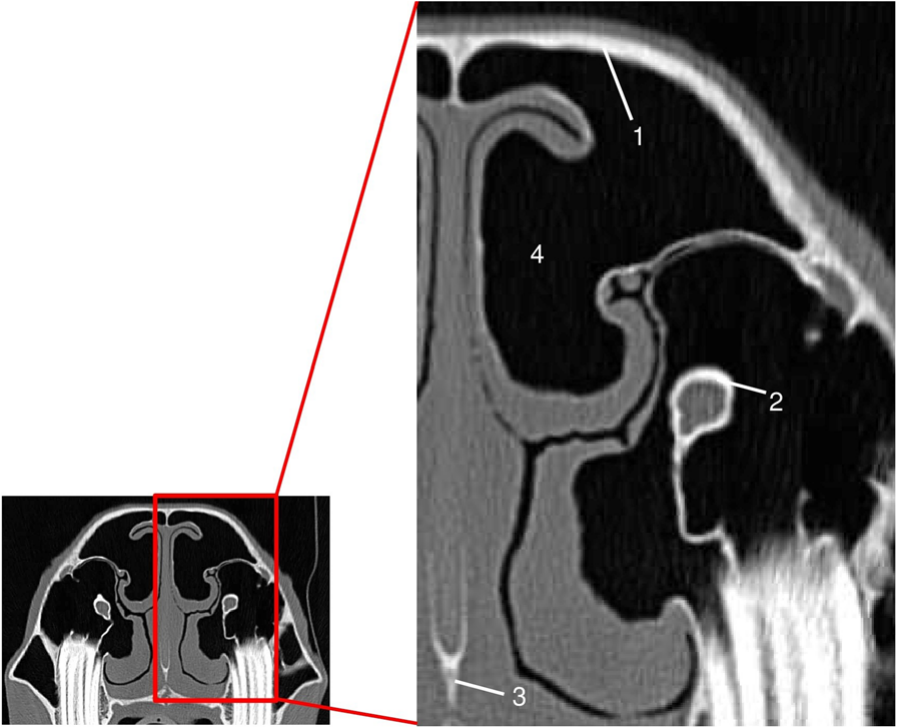
CTbw transversal image in plane 3. (1) frontal bone plate, (2) infraorbital canal, (3) vomer, (4) conchofrontal sinus

When comparing the two CT window presets no significant difference in contour distinction was present (Fig. 4). Nevertheless, both CT window presets were significantly higher rated for the contour distinction of the vomer and the infraorbital canal in comparison to T2w and PDw, whereas no significant difference was present in contour distinction of the nasal and frontal bone plate. T2w and PDw showed no significant differences in all three evaluated locations. T1w was rated significantly lower than all imaging techniques in all evaluated locations (Table 6).

**Table 6:** Median of scoring results of all 3 evaluators for cortical bone appearance

Imaging technique	CTbw		CTstw		T2w		T1w		PDw		score
anatomical parameter	m	%	m	%	m	%	m	%	m	%	
frontal and nasal bone plate	3	0	3	0	2	1.9	1	14.1	2	9.0	0
		0		5.1		26.9		70.5		24.4	1
		41.0		37.2		43.6		15.4		42.3	2
		59.0		57.7		27.6		0		24.4	3
vomer	3	0	2	0	2	3.8	1	33.3	2	14.1	0
		2.6		6.4		33.3		60.3		28.2	1
		34.6		44.9		32.1		6.4		35.9	2
		62.8		48.7		30.8		0		21.8	3
infraorbital canal	3	0	3	0	2	2.6	0	74.4	2	4.3	0
		0		0.9		40.2		24.8		38.5	1
		0.9		47.0		37.6		0.9		40.2	2
		99.1		52.1		19.7		0		17.1	3

m = median; % = distribution of awarded scores (0, 1, 2, 3) in percent

### Resolution of anatomical detail

The detail resolution for cancellous bone was lowest rated in CTstw and T1w. No significant difference was detected between these two techniques in terms of detail resolution. When compared amongst each other, CTbw, T2w and PDw showed no significant difference in rating of detail resolution (Table 7).

**Table 7:** Median of scoring results of all 3 evaluators for detail resolution

Imaging technique	CTbw		CTstw		T2w		T1w		PDw		score
anatomical parameter	m	%	m	%	m	%	m	%	m	%	
cancellous bone	3	0	1	0	3	7.7	2	0	3	0	0
		0		56.4		2.6		41.0		17.9	1
		46.2		43.6		30.8		51.3		30.8	2
		53.8		0		59.0		7.7		51.3	3
ethmoid bone	3	0	1	2.6	2	0	1	5.1	2	15.4	0
		2.6		48.7		7.7		92.3		25.6	1
		43.6		35.9		53.8		2.6		53.8	2
		53.8		12.8		38.5		0		5.1	3
lumen of the nasomaxillary aperture	2	3.8	1	50.0	1	33.3	0	80.8	1	35.9	0
		15.4		34.6		57.7		19.2		53.8	1
		46.2		14.1		9.0		0		6.4	2
		34.6		1.3		0		0		3.8	3

m = median; % = distribution of awarded scores (0, 1, 2, 3) in percent

**Fig. 5:**
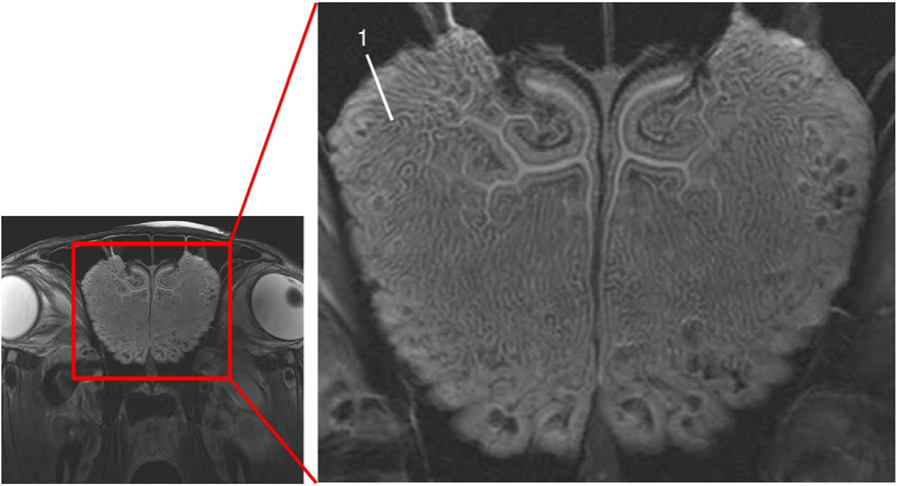
T2w transversal image in plane 1; (1) ethmoid bone

Detail resolution for the ethmoid bone was rated with no significant difference in CTbw, CTstw, T2w (Fig. 5) and PDw. Tw1 was rated significantly lower than all other imaging techniques (Table 7).

**Fig. 6:**
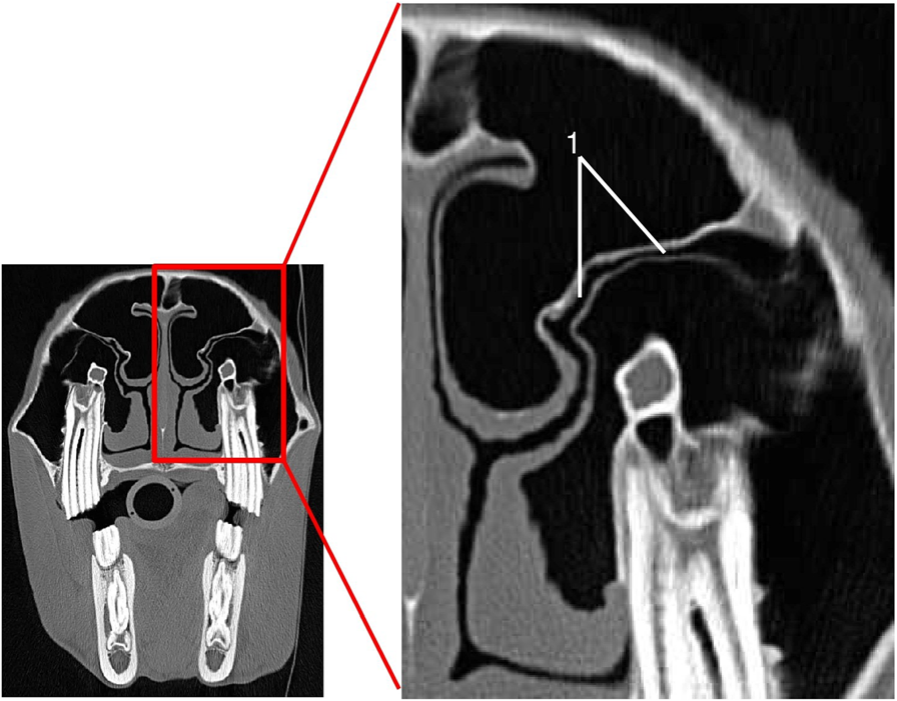
CTbw transversal image in plane 3; (1) nasomaxillary aperture

For the lumen of the nasomaxillary aperture the CTbw was the highest rated imaging modality (Fig. 6). All other imaging techniques showed no significant difference in detail resolution (Table 7).

### Tissue distinction

**Fig. 7:**
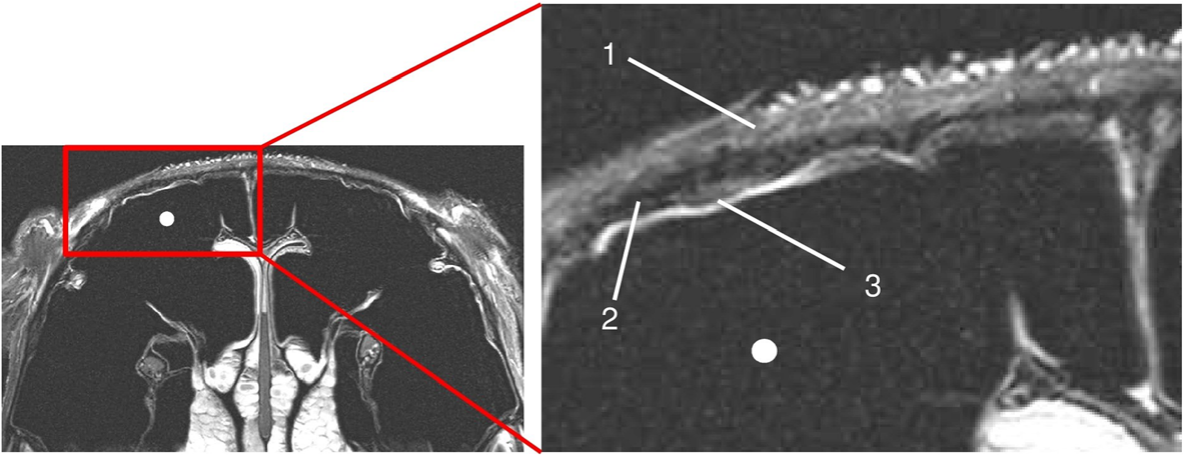
T2w transversal image in plane 2; conchofrontal sinus (white spot); skin (1), signal free frontal bone plate (2), thin bright mucosa (3)

For the distinction between air and mucosal lining, between mucosal lining and cortical bone and between the venous plexus and cortical bone or cartilage (Fig. 7), T2w and PDw showed no significant difference. However, both techniques were significantly superior to the other imaging techniques. T1w was the lowest-rated MRI sequence for all three features. CTbw and CTstw were rated significantly lower when compared to all MRI sequences for the distinction between air and mucosal lining as well as for the distinction between mucosal lining and cortical bone. Nevertheless, no significant difference between the bone and soft tissue window was detected in all three features. Additionally, CTbw, CTstw and T1w showed no significant difference in visualizing the distinction between cortical bone or cartilage and venous plexus.

The distinction between bone and cartilage was evaluated based on the distinction between the vomer and the cartilage of the nasal septum. Here, the median for all imaging techniques lay between 1 and 3 with no statistically significant differences between the five imaging techniques (Table 8).

**Table 8:** Median of scoring results of all 3 evaluators for tissue distinction

Imaging technique	CTbw		CTstw		T2w		T1w		PDw		score
distinction between …	m	%	m	%	m	%	m	%	m	%	
… air and mucosal lining of the rostral maxillary and conchofrontal sinus	0	98.1	0	100	3	0	1	14.7	3	9.6	0
		1.3		0		9.6		61.5		7.1	1
		0.6		0		28.2		23.7		25.6	2
		0		0		62.2		0		57.7	3
… mucosal lining and cortical bone of the rostral maxillary and conchofrontal sinus	0	98.1	0	100	3	0.6	1	15.4	2	8.3	0
		1.9		0		10.3		70.5		10.9	1
		0		0		39.1		14.1		35.9	2
		0		0		50.0		0		44.9	3
… the venous plexus of the nasal septum and cortical bone or cartilage	2	7.1	2	1.9	3	0	2	1.3	3	0	0
		36.5		37.8		1.3		32.1		3.8	1
		45.5		48.1		9		66.7		17.9	2
		10.9		12.2		89.7		0		78.2	3
… bone and cartilage of vomer and nasal septum	2	0	2	0	3	0	1	12.8	3	2.6	0
		25.6		12.8		10.3		51.3		17.9	1
		43.6		82.1		25.6		35.9		15.4	2
		30.8		5.1		64.1		0		64.1	3

m = median; % = distribution of awarded scores (0, 1, 2, 3) in percent

### Image quality parameters

CTbw and CTstw showed significantly less image noise than the MRI sequences. However, no significant difference between the two CT presets was present. The image quality of the T2w sequence was significantly better than that of the other two MRI sequences, which showed no significant difference when compared amongst each other (Table 9).

**Table 9:** Median of scoring results of all 3 evaluators for image quality parameters

imaging technique	CTbw		CTstw		T2w		T1w		PDw		score
quality parameter	m	%	m	%	m	%	m	%	m	%	
image noise	3	0	3	0	3	0	2	0.5	2	2.6	0
		0		0		0		36.4		12.8	1
		2.6		0.5		28.7		53.8		51.3	2
		97.4		99.5		71.3		9.2		33.3	3
image sharpness	3	0	3	0	3	0.5	1	1.0	2	2.1	0
		0		4.6		5.6		81.5		10.3	1
		0		42.6		21.5		17.4		48.2	2
		100		52.8		72.3		0		39.5	3
image contrast	3	0	2	0	3	0	2	0.5	2	1.5	0
		0		3.6		2.6		318		8.7	1
		21		48.2		19		62.6		42.6	2
		79		48.2		78.5		5.1		47.2	3

m = median; % = distribution of awarded scores (0, 1, 2, 3) in percent

The CTbw showed the significantly highest image sharpness of all imaging techniques followed by the CTstw and T2w, which showed no significant difference when compared amongst each other. PDw showed significantly lower image sharpness than the previous three techniques but was nevertheless significantly better than the T1w sequence (Table 9).

The T2w MRI sequence showed the significantly highest image contrast compared to all other imaging techniques. CTbw and CTstw showed no significant difference when compared amongst each other but produced a higher image contrast than the PDw. T1w showed the significantly lowest contrast of all imaging techniques (Table 9).

### Rater agreement

Calculation of inter-rater agreement and internal symmetry revealed that CT examinations showed a higher inter-rater agreement than MRI examinations (Table 10) with a Kappa of 0.7019 for CTbw and 0.6353 for CTstw while T2w, T1w and PDw only showed a Kappa of 0.3339, 0.3704 and 0.333. In all comparisons of the three individual evaluators a significant internal symmetry was present. Nevertheless, these differences and tendencies were more pronounced in the MRI sequences. Rater 3 showed a tendency to evaluate the three MRI sequences with higher scores than the other two raters. Further assessment of those tendencies and differences showed that they did not affect the significance of the inter modality comparison.

**Table 10:** Inter-rater agreement with weighted Kappa (wk) and internal symmetry (p) (p < 0,05) for each modality

Modality		Compared raters			
		rater 1/2	rater 1/3	rater 2/3	all raters
CTbw	p	<0.0001	<0.0001	<0.0166	<0.0148
	wk	0.7999	0.8254	0.9495	0.7019
CTstw	p	<0.0001	<0.0001	<0.0001	<0.0137
	wk	0.7454	0.7072	0.9122	0.6353
T2w	p	0.0016	<0.0001	<0.0001	<0.0158
	wk	0.4815	0.3654	0.5802	0.3339
T1w	p	<0.0001	<0.0001	<0.0001	<0.0153
	wk	0.4855	0.4251	0.5405	0.3704
PDw	p	<0.0001	<0.0001	<0.0001	<0.0141
	wk	0.4629	0.4103	0.6276	0.333

## Discussion

Various studies describe the appearance of the nasal cavities and the paranasal sinuses of the horse either using MRI [14, 16, 21] or CT [22–27]. However, none of these studies implement a direct comparison of CT and MRI under clinical conditions. Furthermore, all of the previous mentioned MRI studies were performed with MRI machines with magnetic field strengths lower than 3.0 T. In the present study a comparison of CT and 3.0 T MRI and their ability to depict different anatomical structures in the anatomical region of the equine nasal cavities and paranasal sinuses was conducted.

The examinations for this study were performed under the same general anaesthesia to reduce the inflicted stress for the horses and lower the risks of general anaesthesia, notably the risk of a second recovery phase [28]. Furthermore, the technique of performing CT of the equine head in standing sedated horses is described [29, 30].

The time needed to scan the region of interest differed strongly between the two techniques. The CT was up to 48 min faster than the MRI. Those differences are due to the two different underlying physical principles of image generation. For the examination of clinical cases veterinarians should consider whether a combined examination of CT and MRI is necessary or if one of the two imaging modalities is more adequate and sufficient for the suspected diagnosis.

In the CT it was possible to scan the entire head in the above-mentioned time frame. The field of view for the MRI examination ranged from 21.7 to 30 cm. The receiver coils used in this study are flexible surface coils that were placed around the structures of interest. However, they only allow a field of view of about 30 cm in length. Slight mispositioning can lead to a loss of signal at the edges of this field and thereby to a loss of quality. For the examination of clinical cases it is therefore important to determine the region of interest beforehand and position the receiver coils accordingly. In this study the caudal edge of the coil was positioned at the level of the zygomatic process.

Transverse images of the head, as acquired in the present study, allow for a good overview of anatomical structures but should not be used as the single means of orientation if pathologies are suspected. A dorsal and sagittal view of the region of interest should be acquired for a better understanding of the three-dimensional spatial relations.

The higher score results for all MRI sequences compared to CT presets in soft tissue imaging are expected results due to the physical basis of image acquisition [31]. The better soft tissue imaging properties of MRI may be of benefit in the diagnostics of tumours and tumour like lesions [17, 31–33]. GERLACH and GERHARDS (2008) described melanoma, adenocarcinoma, neuroendocrine carcinoma, sarcoma, ethmoid hematoma and paranasal cysts with the use of MRI. For detailed diagnostics of the tumour or neoplasia-like lesion, more sequences than those described in this study should be performed to evaluate the extent and quality of the tumour or tumour-like lesion [7, 34]. However, a reliable diagnosis of the quality of the mass can only be achieved by histopathological examination of the tissue.

The detail of cancellous bone was scored with no significant difference for CTbw, T2w, and PDw. In this tissue the strengths of MRI to depict soft tissues and CT to depict bone overlap since the MRI displays the fatty and fluid part of the cancellous bone and the CT displays the thin osseous trabecula.

CT is used in medical imaging diagnostics for its ability to produce a good delineation between soft tissue and bone [23, 31]. In this study, CT presets were scored significantly lower than T2w and PDw sequences for the delineation of mucosal lining and the frontal or nasal bone plate. The mucosal lining of the paranasal sinuses in its healthy state is thin and with low radiodensity so that conventional CT techniques are unable to depict it. MRI is capable of visualizing this soft tissue structure despite its small size and therefore is able to delineate bone and mucosa. In case of a pathologic swelling or hypertrophy of the paranasal mucosa, however, this also becomes visible in conventional CT [9].

T2w and PDw sequences also received higher score values for the depiction of the differences between the venous plexus of the nasal mucosa and the bone or cartilage of the nasal septum. These results can also be attributed to the MRI’s superior ability to depict soft tissue.

In this study a resolution with a matrix of 1024 x 1024 resulted in highly detailed images in T2w MRI. In MRI, the high resolution correlates to the amount of time spent for one sequence [35]. This explains the higher acquisition times for the T2w, which was given the highest score values for image sharpness. The T1w sequence was given the lowest scores for image sharpness, which is consistent with the shortest acquisition times and the lower resolution with a matrix of 720 x 720. Furthermore, not only the resolution but also the image contrast is time-related in MRI. Any amount of time saved in a preset MRI sequence usually results in a loss of quality [35]. These properties may be of importance in clinical cases if delicate structures need to be depicted at high detail.

In this study, T2w, T1w and PDw sequences were chosen for comparison with CT images. These sequences are described to give a good anatomical overview [7, 14, 16]. For T2w and PDw sequences the higher magnetic field strength of the 3 Tesla magnet is advantageous since it allows for a higher signal to noise ratio and higher contrast [35–37]. Only through the properties of the 3.0 T magnet was high resolution imaging made possible in a reasonable timeframe for this study. The T1w sequence was performed as a gradient echo sequence a spin echo (SE) sequence to save time. Nevertheless, one downside of the 3.0 T MRI compared to magnets with lower field strengths is that in T1w SE sequences the time for acquisition is prolonged since the T1-relaxation time is extended [35–37]. T1w was the lowest-rated MRI sequence in the present study. This verifies the disadvantage of the 3.0 T MRI and suggests that clinicians should rather acquire T2w images for orientation and first impressions of a pathological process.

The long examination times in MRI were chosen to create very high quality images to explore the possibilities of 3.0 T magnetic resonance imaging. In clinical use those high resolutions might be reduced to save time, since it is still possible to acquire images with a reasonable quality for diagnostics with shorter examination periods. Should high resolution images be required to evaluate particularly delicate structures, a reduction of the field of view might be another option to save time.

Although the T2w sequence produced images with the same resolution as the CT, the detail resolution of the nasomaxillary aperture was significantly better rated in CTbw than in the T2w. This is explicable through the different special resolutions of the two techniques. The CTbw has a slice thickness of only 1.5 mm compared to 4.0 mm of the T2w. The higher slice thickness leads to the partial volume effect which is an artefact that occurs when the properties of different tissues or structures are averaged in one voxel [35]. This artefact leads to a blurred appearance of delicate structures. If a congestion of the nasomaxillary aperture is suspected, the CTbw is the technique of choice as shown by BRINKSCHULTE (2012).

The graded scoring system applied in this study was designed to objectify the weak-and strongpoints of the resulting images from each imaging technique without focusing solely on technical data and numbers. For this reason, the scoring system relies on the assessment of the images through human validation. Consequently, the deviation in inter-rater agreement resulted from the subjective impression that each evaluator expresses in the awarded scores. Despite these differing impressions, advantages and disadvantages of one imaging technique over the other were measurable. In contrast to the present study, a comparative study of MRI and CT of the equine fetlock joint showed a higher inter-rater agreement [20]. This study also used 3.0 T MRI. The different outcome may result from the more complex anatomy of the nasal cavities and paranasal sinuses, which render the interpretation in MRI more challenging.

## Conclusion

The results of this study suggest that CT is still the imaging technique of choice if osseous structures are involved and short examination times are of importance. CT may allow for a differentiation of bone from soft tissues, but only if soft tissue structures possess a high radiodensity and size.

On the contrary, MRI depicts soft tissue structures in high detail and is able to differentiate between different soft tissue types. A benefit of the high resolution MR images is the ability to depict delicate structures such as the infraorbital canal and its contents so that previously undetected lesions may be visualized. 3.0 T MRI does not abolish the inherent problem of MRI that examinations take up a long time if more sequences and orientations are necessary to assess pathologic processes.

In conclusion, we show that both imaging techniques complement each other and that it is possible to examine horses’ heads in CT and MRI under the same general anaesthesia without complications. A surgical intervention under the same general anaesthesia, however, will seldom be possible due to the prolonged time required for preparation and surgery.

## Abbreviations

3D: Three dimensional
CT: Computed tomography
CTbw: CT image with bone window
CTstw: CT image with soft tissue window
ISS: Inter-slice spacing
MRI: Magnetic resonance imaging
NSA: Number of signal averages
PDw: Proton density weighted
SD: Standard deviation
SE: Spin echo sequence
ST: Slice thickness
T1w: T1 weighted
T2w: T2 weighted
TE: Echo time
TR: Repetition time
WL: Window level
WW: Window width
